# Supplementary material for: Empathy training for service employees: A mixed-methods systematic review
Source: PLoS One. 2023 Aug 14;18(8):e0289793. doi: 10.1371/journal.pone.0289793 (PMC10424876; doi:10.1371/journal.pone.0289793)
Supplement: S1 Appendix — (DOCX) [file pone.0289793.s002.docx]

**S1 Appendix. The search strategy**

|  | **ABI/Inform Global** | Initial search strategy | Updated search strategy |
| --- | --- | --- | --- |
|  |  | August 6, 2019 | August 7th, 2022 to April 1st, 2022 |
| S1 | MAINSUBJECT.EXACT("Virtual reality") OR MAINSUBJECT.EXACT("Experiential learning") OR MAINSUBJECT.EXACT("Simulation") OR MAINSUBJECT.EXACT("Training") OR MAINSUBJECT.EXACT("Teaching methods") OR MAINSUBJECT.EXACT("Storytelling") OR MAINSUBJECT.EXACT("Teaching") OR MAINSUBJECT.EXACT("Education") OR MAINSUBJECT.EXACT("Role playing") | 149 119 | 35 251 |
| S2 | ab(training OR teaching OR courses OR "education methods" OR "educational methodologies" OR "experiential learning" OR "scenario techniques" OR simulat* OR "role play" OR "role playing" OR "virtual reality" OR storytelling) | 475 625 | 4 758 |
| S3 | ti(training OR teaching OR courses OR "education methods" OR "educational methodologies" OR "experiential learning" OR "Scenario techniques" OR Simulat* OR "role play" OR "role playing" OR "virtual reality" OR storytelling) | 101 912 | 11 266 |
| S4 | S1 OR S2 OR S3 | 587 378 | 75 459 |
| S5 | MAINSUBJECT.EXACT("Workers") OR MAINSUBJECT.EXACT("Psychologists") OR MAINSUBJECT.EXACT("Health services") OR MAINSUBJECT.EXACT("Teachers") OR MAINSUBJECT.EXACT("Customer services") OR MAINSUBJECT.EXACT("Services") OR MAINSUBJECT.EXACT("Educational services") OR MAINSUBJECT.EXACT("Service industries") OR MAINSUBJECT.EXACT("Business services") OR MAINSUBJECT.EXACT("Physicians") OR MAINSUBJECT.EXACT("Caregivers") OR MAINSUBJECT.EXACT("Nurses") OR MAINSUBJECT.EXACT("Employees") OR MAINSUBJECT.EXACT("Staff") OR MAINSUBJECT.EXACT("Therapists") OR MAINSUBJECT.EXACT("Health care industry") | 322 375 | 138 036 |
| S6 | ab("service Employees" OR "service Personnel" OR "service staff" OR "service workers" OR "service organizations" OR "service organisations" OR "educational services" OR "business services" OR "customer services" OR "health services" OR Caregivers OR Carers OR therapists OR counsel* OR doctors OR "healthcare providers" OR Nurses OR Physicians OR Psychologists) | 195 768 | 1 436 |
| S7 | ti("service Employees" OR "service Personnel" OR "service staff" OR "service workers" OR "service organizations" OR "service organisations" OR "educational services" OR "business services" OR "customer services" OR "health services" OR Caregivers OR Carers OR therapists OR counsel* OR doctors OR "healthcare providers" OR Nurses OR Physicians OR Psychologists) | 51 613 | 8 096 |
| S8 | S5 OR S6 OR S7 | 488 063 | 148 351 |
| S9 | MAINSUBJECT.EXACT("Altruism") OR MAINSUBJECT.EXACT("Sympathy") OR MAINSUBJECT.EXACT("Emotional intelligence") OR MAINSUBJECT.EXACT("Empathy") | 4 066 | 2 604 |
| S10 | ab(Empath* OR Altruis* OR Compassion* OR Sympath* OR "emotional intelligence" OR "emotional connection" OR "emotional contagion" OR "Helping behavior" OR "Helping behaviour" OR "Helping attitude" OR "active Listening" OR "Prosocial behavior" OR "Prosocial behaviour" OR "Prosocial attitude" OR "Pro-social behavior" OR "Pro-social behaviour" OR "Pro-social attitude" OR "Theory of mind" OR "Understanding of others") | 31 011 | 3 822 |
| S11 | ti(Empath* OR Altruis* OR Compassion* OR Sympath* OR "emotional intelligence" OR "emotional connection" OR "emotional contagion" OR "Helping behavior" OR "Helping behaviour" OR "Helping attitude" OR "active Listening" OR "Prosocial behavior" OR "Prosocial behaviour" OR "Prosocial attitude" OR "Pro-social behavior" OR "Pro-social behaviour" OR "Pro-social attitude" OR "Theory of mind" OR "Understanding of others") | 6 959 | 1 666 |
| S12 | S9 OR S10 OR S11 | 35 094 | 5 468 |
| S13 | S4 AND S8 AND S12 | 534 | 246 |

|  | **Business Source Premier** | Initial search strategy | Updated search strategy |
| --- | --- | --- | --- |
|  |  | August 6, 2019 | August 7th, 2022 to April 1st, 2022 |
| S1 | DE "TRAINING" OR DE "SIMULATION methods & models" OR DE "OCCUPATIONAL training" OR DE "STORYTELLING in business" | 7 508 | 3 965 |
| S2 | AB training OR teaching OR courses OR "education methods" OR "educational methodologies" OR "experiential learning" OR "Scenario techniques" OR Simulat* OR "role play" OR "role playing" OR "virtual reality" OR storytelling | 503 471 | 86 576 |
| S3 | TI training OR teaching OR courses OR "education methods" OR "educational methodologies" OR "experiential learning" OR "Scenario techniques" OR Simulat* OR "role play" OR "role playing" OR "virtual reality" OR storytelling | 103 310 | 72 919 |
| S4 | S1 OR S2 OR S3 | 541 688 | 89 447 |
| S5 | DE "EMPLOYEES" OR DE "MEDICAL personnel" OR DE "PROFESSIONAL employees" OR DE "SERVICE industries workers" OR DE "HEALTH care industry" OR DE "NURSES" OR DE "PHYSICIANS" OR DE "PSYCHOLOGISTS" OR DE "TEACHERS" OR DE "CUSTOMER services" | 264 713 | 28 909 |
| S6 | AB "service Employees" OR "service Personnel" OR "service staff" OR "service workers" OR "service organizations" OR "service organisations" OR "educational services" OR "business services" OR "customer services" OR "health services" OR Caregivers OR Carers OR therapists OR counsel* OR doctors OR "healthcare providers" OR Nurses OR Physicians OR Psychologists | 195 412 | 41 211 |
| S7 | TI "service Employees" OR "service Personnel" OR "service staff" OR "service workers" OR "service organizations" OR "service organisations" OR "educational services" OR "business services" OR "customer services" OR "health services" OR Caregivers OR Carers OR therapists OR counsel* OR doctors OR "healthcare providers" OR Nurses OR Physicians OR Psychologists | 40 786 | 5 673 |
| S8 | S5 OR S6 OR S7 | 433 958 | 59 394 |
| S9 | DE "ACTIVE listening" OR DE "EMOTIONAL labor" | 610 | 319 |
| S10 | AB Empath* OR Altruis* OR Compassion* OR Sympath* OR "emotional intelligence" OR "emotional connection" OR "emotional contagion" OR "Helping behavior" OR "Helping behaviour" OR "Helping attitude" OR "active Listening" OR "Prosocial behavior" OR "Prosocial behaviour" OR "Prosocial attitude" OR "Pro-social behavior" OR "Pro-social behaviour" OR "Pro-social attitude" OR "Theory of mind" OR "Understanding of others" | 21 675 | 5 619 |
| S11 | TI Empath* OR Altruis* OR Compassion* OR Sympath* OR "emotional intelligence" OR "emotional connection" OR "emotional contagion" OR "Helping behavior" OR "Helping behaviour" OR "Helping attitude" OR "active Listening" OR "Prosocial behavior" OR "Prosocial behaviour" OR "Prosocial attitude" OR "Pro-social behavior" OR "Pro-social behaviour" OR "Pro-social attitude" OR "Theory of mind" OR "Understanding of others" | 6 368 | 4 856 |
| S12 | S9 OR S10 OR S11 | 24 326 | 6 086 |
| S13 | S4 AND S8 AND S12 | 252 | 103 |

|  | **Cinhal plus with Fulltext** | Initial search strategy | Updated search strategy |
| --- | --- | --- | --- |
|  |  | August 6, 2019 | August 7th, 2022 to April 1st, 2022 |
| S1 | (MM "Teaching") | 4 201 | 349 |
| S2 | (MM "Education") | 4 101 | 269 |
| S3 | (MM "Teaching Methods") | 15 787 | 2 445 |
| S4 | (MM "Learning Methods") | 6 465 | 2 453 |
| S5 | (MM "Simulations") | 5 981 | 2 015 |
| S6 | (MM "Experiential Learning") | 1 135 | 225 |
| S7 | (MM "Role Playing") | 806 | 69 |
| S8 | (MM "Virtual Reality") | 2 798 | 1 450 |
| S9 | (MM "Storytelling") | 1 645 | 405 |
| S10 | S1 OR S2 OR S3 OR S4 OR S5 OR S6 OR S7 OR S8 OR S9 | 40 488 | 8 708 |
| S11 | AB training OR teaching OR courses OR "education methods" OR "educational methodologies" OR "experiential learning" OR "Scenario techniques" OR Simulat* OR "role play" OR "role playing" OR "virtual reality" OR storytelling | 270 438 | 94 432 |
| S12 | TI training OR teaching OR courses OR "education methods" OR "educational methodologies" OR "experiential learning" OR "Scenario techniques" OR Simulat* OR "role play" OR "role playing" OR "virtual reality" OR storytelling | 99 453 | 67 639 |
| S13 | S9 OR S10 OR S11 | 342 296 | 98 584 |
| S14 | (MM "Employee Attitudes") | 2 072 | 514 |
| S15 | (MM "Health Personnel") | 18 127 | 6 046 |
| S16 | (MM "Health Services") | 7 221 | 1 173 |
| S17 | (MM "Health Care Delivery") | 28 769 | 6 186 |
| S18 | (MM "Health Care Industry") | 6 434 | 1 031 |
| S19 | (MM "Nurses") | 33 359 | 3 930 |
| S20 | (MM "Caregivers") | 19 207 | 4 215 |
| S21 | (MM "Physicians") | 27 558 | 3 794 |
| S22 | (MM "Psychologists") | 1 716 | 549 |
| S23 | (MM "Teachers") | 5 146 | 976 |
| S24 | S14 OR S15 OR S16 OR S17 OR S18 OR S19 OR S20 OR S21 OR S22 OR S23 | 146 069 | 28 |
| S25 | AB "service Employees" OR "service Personnel" OR "service staff" OR "service workers" OR "service organizations" OR "service organisations" OR "educational services" OR "business services" OR "customer services" OR "health services" OR Caregivers OR Carers OR therapists OR counsel* OR doctors OR "healthcare providers" OR Nurses OR Physicians OR Psychologists | 418 505 | 168 964 |
| S26 | TI "service Employees" OR "service Personnel" OR "service staff" OR "service workers" OR "service organizations" OR "service organisations" OR "educational services" OR "business services" OR "customer services" OR "health services" OR Caregivers OR Carers OR therapists OR counsel* OR doctors OR "healthcare providers" OR Nurses OR Physicians OR Psychologists | 167 925 | 168 955 |
| S27 | S24 OR S25 OR S26 | 615 662 | 177 483 |
| S28 | (MM "Empathy") | 3 780 | 1 027 |
| S29 | (MM "Compassion") | 517 | 645 |
| S30 | (MM "Caring") | 5 218 | 353 |
| S31 | (MM "Altruism") | 908 | 145 |
| S32 | (MM "Theory of Mind") | 448 | 240 |
| S33 | (MM "Listening") | 1 265 | 239 |
| S34 | (MM "Emotional Intelligence") | 1 277 | 344 |
| S35 | S28 OR S29 OR S30 OR S31 OR S32 OR S33 OR S34 | 13 125 | 2 875 |
| S36 | AB Empath* OR Altruis* OR Compassion* OR Sympath* OR "emotional intelligence" OR "emotional connection" OR "emotional contagion" OR "Helping behavior" OR "Helping behaviour" OR "Helping attitude" OR "active Listening" OR "Prosocial behavior" OR "Prosocial behaviour" OR "Prosocial attitude" OR "Pro-social behavior" OR "Pro-social behaviour" OR "Pro-social attitude" OR "Theory of mind" OR "Understanding of others" | 22 940 | 9 763 |
| S37 | TI Empath* OR Altruis* OR Compassion* OR Sympath* OR "emotional intelligence" OR "emotional connection" OR "emotional contagion" OR "Helping behavior" OR "Helping behaviour" OR "Helping attitude" OR "active Listening" OR "Prosocial behavior" OR "Prosocial behaviour" OR "Prosocial attitude" OR "Pro-social behavior" OR "Pro-social behaviour" OR "Pro-social attitude" OR "Theory of mind" OR "Understanding of others" | 10 923 | 8 205 |
| S38 | (S35 OR S36 OR S37) | 36 235 | 10 544 |
| S39 | (S13 AND S27 AND S38) | 1 832 | 822 |

|  | **Embase** | Initial search strategy | Updated search strategy |
| --- | --- | --- | --- |
|  |  | August 6, 2019 | August 7th, 2022 to April 1st, 2022 |
| #1 | training'/mj | 20 416 | 2 449 |
| #2 | teaching'/mj | 37 286 | 3 751 |
| #3 | education'/mj | 82 825 | 14 040 |
| #4 | 'educational technology'/mj | 1 065 | 148 |
| #5 | 'experiential learning'/mj | 391 | 186 |
| #6 | 'simulation training'/mj | 1 761 | 1 813 |
| #7 | 'role playing'/mj | 5 631 | 182 |
| #8 | 'virtual reality'/mj | 6 570 | 4 046 |
| #9 | 'sensitivity training'/mj | 147 | 9 |
| #10 | 'storytelling'/mj | 390 | 163 |
| #11 | training:ab,ti OR teaching:ab,ti OR courses:ab,ti OR 'education methods':ab,ti OR 'educational methodologies':ab,ti OR 'experiential learning':ab,ti OR 'scenario techniques':ab,ti OR simulat*:ab,ti OR 'role play':ab,ti OR 'role playing':ab,ti OR 'virtual reality':ab,ti OR storytelling:ab,ti | 1 213 362 | 339 331 |
| #12 | #1 OR #2 OR #3 OR #4 OR #5 OR #6 OR #7 OR #8 OR #9 OR #10 OR #11 | 1 295 247 | 348 927 |
| #13 | 'personnel'/mj | 4 630 | 475 |
| #14 | 'staff'/mj | 2 192 | 182 |
| #15 | 'worker'/mj | 4 676 | 1 457 |
| #16 | 'health care personnel'/mj | 26 126 | 8 809 |
| #17 | 'health service'/mj | 54 102 | 3 461 |
| #18 | 'health care industry'/mj | 1 018 | 115 |
| #19 | 'nurse'/mj | 44 008 | 6 495 |
| #20 | 'caregiver'/mj | 20 046 | 6 064 |
| #21 | 'physician'/mj | 58 434 | 7 366 |
| #22 | 'psychologist'/mj | 921 | 173 |
| #23 | 'teacher'/mj | 7 610 | 1 270 |
| #24 | service employees':ab,ti OR 'service personnel':ab,ti OR 'service staff':ab,ti OR 'service workers':ab,ti OR 'service organizations':ab,ti OR 'service organisations':ab,ti OR 'educational services':ab,ti OR 'business services':ab,ti OR 'customer services':ab,ti OR 'health services':ab,ti OR caregivers:ab,ti OR carers:ab,ti OR therapists:ab,ti OR counsel*:ab,ti OR doctors:ab,ti OR 'healthcare providers':ab,ti OR nurses:ab,ti OR physicians:ab,ti OR psychologists:ab,ti | 906 589 | 203 683 |
| #25 | #13 OR #14 OR #15 OR #16 OR #17 OR #18 OR #19 OR #20 OR #21 OR #22 OR #23 OR #24 | 1 040 055 | 219 492 |
| #26 | 'empathy'/mj | 9 503 | 2 074 |
| #27 | 'altruism'/mj | 2 597 | 361 |
| #28 | 'compassion'/mj | 62 | 20 |
| #29 | 'theory of mind'/mj | 1 754 | 607 |
| #30 | 'comprehension'/mj | 6 145 | 1 183 |
| #31 | 'caring'/mj | 1 | 1 |
| #32 | 'listening'/mj | 60 | 2 |
| #33 | 'sympathy'/mj | 39 | 2 |
| #34 | 'prosocial behavior'/mj | 43 | 128 |
| #35 | 'emotional intelligence'/mj | 1 447 | 524 |
| #36 | empath*:ab,ti OR altruis*:ab,ti OR compassion*:ab,ti OR sympath*:ab,ti OR 'emotional intelligence':ab,ti OR 'emotional connection':ab,ti OR 'emotional contagion':ab,ti OR 'helping behavior':ab,ti OR 'helping behaviour':ab,ti OR 'helping attitude':ab,ti OR 'active listening':ab,ti OR 'prosocial behavior':ab,ti OR 'prosocial behaviour':ab,ti OR 'prosocial attitude':ab,ti OR 'pro-social behavior':ab,ti OR 'pro-social behaviour':ab,ti OR 'pro-social attitude':ab,ti OR 'theory of mind':ab,ti OR 'understanding of others':ab,ti | 167 961 | 24 436 |
| #37 | #26 OR #27 OR #28 OR #29 OR #30 OR #31 OR #32 OR #33 OR #34 OR #35 OR #36 | 179 245 | 27 296 |
| #38 | S12 AND S25 AND S37 | 2 710 | 1 184 |

|  | **ERIC** | Initial search strategy | Updated search strategy |
| --- | --- | --- | --- |
|  |  | August 6, 2019 | August 7th, 2022 to April 1st, 2022 |
| S1 | DE "Training" OR DE "Education" OR DE "Instruction" OR DE "Training Methods" OR DE "Teaching Methods" OR DE "Sensitivity Training" OR DE "Role Playing" OR DE "Simulation" OR DE "Experiential Learning" | 235 962 | 17 753 |
| S2 | AB training OR teaching OR courses OR "education methods" OR "educational methodologies" OR "experiential learning" OR "Scenario techniques" OR Simulat* OR "role play" OR "role playing" OR "virtual reality" OR storytelling | 465 467 | 4 255 |
| S3 | TI training OR teaching OR courses OR "education methods" OR "educational methodologies" OR "experiential learning" OR "Scenario techniques" OR Simulat* OR "role play" OR "role playing" OR "virtual reality" OR storytelling | 146 739 | 39 772 |
| S4 | S1 OR S2 OR S3 | 592 126 | 42 859 |
| S5 | DE "Employees" OR DE "Health Personnel" OR DE "Health Services" OR DE "Professional Services" OR DE "Nurses" OR DE "Caregivers" OR DE "Physicians" OR DE "Psychologists" OR DE "Teachers" | 37 575 | 1 384 |
| S6 | AB "service Employees" OR "service Personnel" OR "service staff" OR "service workers" OR "service organizations" OR "service organisations" OR "educational services" OR "business services" OR "customer services" OR "health services" OR Caregivers OR Carers OR therapists OR counsel* OR doctors OR "healthcare providers" OR Nurses OR Physicians OR Psychologists | 87 803 | 4 881 |
| S7 | TI "service Employees" OR "service Personnel" OR "service staff" OR "service workers" OR "service organizations" OR "service organisations" OR "educational services" OR "business services" OR "customer services" OR "health services" OR Caregivers OR Carers OR therapists OR counsel* OR doctors OR "healthcare providers" OR Nurses OR Physicians OR Psychologists | 28 520 | 4 878 |
| S8 | S5 OR S6 OR S7 | 115 679 | 5 418 |
| S9 | DE "Empathy" OR DE "Caring" OR DE "Altruism" OR DE "Helping Relationship" OR DE "Theory of Mind" OR DE "Prosocial Behavior" OR DE "Emotional Intelligence" | 15 961 | 2 051 |
| S10 | AB Empath* OR Altruis* OR Compassion* OR Sympath* OR "emotional intelligence" OR "emotional connection" OR "emotional contagion" OR "Helping behavior" OR "Helping behaviour" OR "Helping attitude" OR "active Listening" OR "Prosocial behavior" OR "Prosocial behaviour" OR "Prosocial attitude" OR "Pro-social behavior" OR "Pro-social behaviour" OR "Pro-social attitude" OR "Theory of mind" OR "Understanding of others" | 11 712 | 1 792 |
| S11 | TI Empath* OR Altruis* OR Compassion* OR Sympath* OR "emotional intelligence" OR "emotional connection" OR "emotional contagion" OR "Helping behavior" OR "Helping behaviour" OR "Helping attitude" OR "active Listening" OR "Prosocial behavior" OR "Prosocial behaviour" OR "Prosocial attitude" OR "Pro-social behavior" OR "Pro-social behaviour" OR "Pro-social attitude" OR "Theory of mind" OR "Understanding of others" | 3 284 | 1 373 |
| S12 | S9 OR S10 OR S11 | 21 628 | 2 457 |
| S13 | S4 AND S8 AND S12 | 1 120 | 84 |

|  | **Medline** | Initial search strategy | Updated search strategy |
| --- | --- | --- | --- |
|  |  | August 6, 2019 | August 7th, 2022 to April 1st, 2022 |
| 1 | (Teaching or Simulation training or Education or Educational technology or Role playing or Virtual reality or Sensitivity training groups).sh. | 73 904 | 7 344 |
| 2 | (training or teaching or courses or "education methods" or "educational methodologies" or "experiential learning" or "Scenario techniques" or Simulat* or "role play" or "role playing" or "virtual reality" or storytelling).ab. | 895 873 | 227 780 |
| 3 | (training or teaching or courses or "education methods" or "educational methodologies" or "experiential learning" or "Scenario techniques" or Simulat* or "role play" or "role playing" or "virtual reality" or storytelling).ti. | 250 982 | 47 690 |
| 4 | 1 or 2 or 3 | 1 030 119 | 238 265 |
| 5 | (Health personnel or Health services or Health care sector or Physicians or Nurses or Caregivers or General practitioners or Psychology).sh. | 244 687 | 43 196 |
| 6 | ("service Employees" or "service Personnel" or "service staff" or "service workers" or "service organizations" or "service organisations" or "educational services" or "business services" or "customer services" or "health services" or Caregivers or Carers or therapists or counsel* or doctors or "healthcare providers" or Nurses or Physicians or Psychologists).ab. | 579 072 | 120 543 |
| 7 | ("service Employees" or "service Personnel" or "service staff" or "service workers" or "service organizations" or "service organisations" or "educational services" or "business services" or "customer services" or "health services" or Caregivers or Carers or therapists or counsel* or doctors or "healthcare providers" or Nurses or Physicians or Psychologists).ti. | 191 913 | 25 146 |
| 8 | 5 or 6 or 7 | 834 676 | 143 909 |
| 9 | (Empathy or Altruism or Comprehension or Theory of mind or Helping behavior or Emotional intelligence).sh. | 43 306 | 6 865 |
| 10 | (Empath* or Altruis* or Compassion* or Sympath* or "emotional intelligence" or "emotional connection" or "emotional contagion" or "Helping behavior" or "Helping behaviour" or "Helping attitude" or "active Listening" or "Prosocial behavior" or "Prosocial behaviour" or "Prosocial attitude" or "Pro-social behavior" or "Pro-social behaviour" or "Pro-social attitude" or "Theory of mind" or "Understanding of others").ab. | 111 609 | 16 178 |
| 11 | (Empath* or Altruis* or Compassion* or Sympath* or "emotional intelligence" or "emotional connection" or "emotional contagion" or "Helping behavior" or "Helping behaviour" or "Helping attitude" or "active Listening" or "Prosocial behavior" or "Prosocial behaviour" or "Prosocial attitude" or "Pro-social behavior" or "Pro-social behaviour" or "Pro-social attitude" or "Theory of mind" or "Understanding of others").ti. | 48 444 | 5 270 |
| 12 | 9 OR 10 OR 11 | 161 459 | 20 108 |
| 13 | 4 AND 8 AND 12 | 2 433 | 699 |

|  | **PsychInfo** | Initial search strategy | Updated search strategy |
| --- | --- | --- | --- |
|  |  | August 6, 2019 | August 7th, 2022 to April 1st, 2022 |
| 1 | (Training or Teaching or Education or Personnel training or Sensitivity training or Teaching methods or Experiential learning or Learning strategies or Simulation or Virtual reality or Role playing or Social emotional learning or Storytelling).sh. | 169 944 | 30 186 |
| 2 | (training or teaching or courses or "education methods" or "educational methodologies" or "experiential learning" or "Scenario techniques" or Simulat* or "role play" or "role playing" or "virtual reality" or storytelling).ab. | 411 854 | 57 266 |
| 3 | (training or teaching or courses or "education methods" or "educational methodologies" or "experiential learning" or "Scenario techniques" or Simulat* or "role play" or "role playing" or "virtual reality" or storytelling).ti. | 113 358 | 13 911 |
| 4 | 1 or 2 or 3 | 509 155 | 69 367 |
| 5 | (Professional personnel or Health personnel or Medical personnel or Personnel or Health care services or Caregivers or Nurses or Physicians or Allied health personnel or Clinicians or Therapists or Psychologists or General practitioners or Service personnel or Business) and industrial personnel) or Educational personnel).sh. | 10 994 | 1 321 |
| 6 | ("service Employees" or "service Personnel" or "service staff" or "service workers" or "service organizations" or "service organisations" or "educational services" or "business services" or "customer services" or "health services" or Caregivers or Carers or therapists or counsel* or doctors or "healthcare providers" or Nurses or Physicians or Psychologists).ab. | 350 862 | 44 798 |
| 7 | ("service Employees" or "service Personnel" or "service staff" or "service workers" or "service organizations" or "service organisations" or "educational services" or "business services" or "customer services" or "health services" or Caregivers or Carers or therapists or counsel* or doctors or "healthcare providers" or Nurses or Physicians or Psychologists).ti. | 92 733 | 11 764 |
| 8 | 5 or 6 or 7 | 377 741 | 46 804 |
| 9 | (Empathy or Altruism or Sympathy or Comprehension or Prosocial behavior or Theory of mind or Caring behaviors or Listening Interpersonal or Emotion recognition or Emotional intelligence or Assistance Social Behavior).sh. | 53 160 | 10 277 |
| 10 | (Empath* or Altruis* or Compassion* or Sympath* or "emotional intelligence" or "emotional connection" or "emotional contagion" or "Helping behavior" or "Helping behaviour" or "Helping attitude" or "active Listening" or "Prosocial behavior" or "Prosocial behaviour" or "Prosocial attitude" or "Pro-social behavior" or "Pro-social behaviour" or "Pro-social attitude" or "Theory of mind" or "Understanding of others").ab. | 74 244 | 12 913 |
| 11 | (Empath* or Altruis* or Compassion* or Sympath* or "emotional intelligence" or "emotional connection" or "emotional contagion" or "Helping behavior" or "Helping behaviour" or "Helping attitude" or "active Listening" or "Prosocial behavior" or "Prosocial behaviour" or "Prosocial attitude" or "Pro-social behavior" or "Pro-social behaviour" or "Pro-social attitude" or "Theory of mind" or "Understanding of others").ti. | 23 885 | 4 802 |
| 12 | 9 or 10 or 11 | 102 012 | 17 584 |
| 13 | 4 and 8 and 12 | 2 567 | 527 |

|  | **Web of science** | Initial search strategy | Updated search strategy |
| --- | --- | --- | --- |
|  |  | August 6, 2019 | August 7th, 2022 to April 1st, 2022 |
| #1 | TOPIC: (training OR teaching OR courses OR "education methods" OR "educational methodologies" OR "experiential learning" OR "Scenario techniques" OR Simulat* OR "role play" OR "role playing" OR "virtual reality" OR storytelling) | 4 913 852 | 1 177 704 |
| #2 | TOPIC: ("service Employees" OR "service Personnel" OR "service staff" OR "service workers" OR "service organizations" OR "service organisations" OR "educational services" OR "business services" OR "customer services" OR "health services" OR Caregivers OR Carers OR therapists OR counsel* OR doctors OR "healthcare providers" OR Nurses OR Physicians OR Psychologists) | 1 009 217 | 245 621 |
| #3 | TOPIC: (Empath* OR Altruis* OR Compassion* OR Sympath* OR "emotional intelligence" OR "emotional connection" OR "emotional contagion" OR "Helping behavior" OR "Helping behaviour" OR "Helping attitude" OR "active Listening" OR "Prosocial behavior" OR "Prosocial behaviour" OR "Prosocial attitude" OR "Pro-social behavior" OR "Pro-social behaviour" OR "Pro-social attitude" OR "Theory of mind" OR "Understanding of others") | 196 885 | 40 076 |
| #4 | #3 AND #2 AND #1 | 3 593 | 1 596 |
