# Supplementary material for: Empathy training for service employees: A mixed-methods systematic review
Source: PLoS One. 2023 Aug 14;18(8):e0289793. doi: 10.1371/journal.pone.0289793 (PMC10424876; doi:10.1371/journal.pone.0289793)
Supplement: Attachment — Submitted filename: PONE-D-22-28102_Response_to_Reviewers.docx [file pone.0289793.s004.docx]

PONE-D-22-28102

Empathy training for service employees: A mixed-methods systematic review

PLOS ONE

>>> Responses to reviewers:

| **Reviewer #1** | **Responses** |
| --- | --- |
| My main concerns about the paper are that it is essentially a systematic review of empathy training in the healthcare setting. Despite being framed as a review of papers in the service industry, only papers relating to healthcare were included. I am unsure if this review adds to the many other systematic reviews of empathy training in healthcare published in recent years.  The authors present more of a narrative review and there is little discussion on the effectiveness of training (which in my mind would be most relevant). I appreciate the authors comment that this is the first review of empathy training programs in the service research – but as there were no papers outside of the healthcare field identified and included – it is not a review of empathy training for service employees but instead a review of empathy-training in healthcare service. | We appreciate the reviewer’s comment and understand their concern about the relevance of our paper as compared to current systematic reviews on empathy training. However, we believe that our work adds value to the existing literature.  First, we intended to look at empathy training in every service domain, including business and marketing, while current empathic training systematic reviews overtly focus on healthcare services. Our search strategy clearly states our intentions to dig into different databases, including business databases, to obtain a more comprehensive view of empathy training in service. The fact that we do not identify papers from services in marketing is an exciting result *per se*. As outlined in our introduction, many prominent services marketing authors praise empathy’s crucial role during service interactions and the importance of training frontline employees on empathy. Our work shows a gap between statements of interest for empathy and empathy training in service research and the actual state of the literature, which show no empirical paper testing empathy training in business contexts. It also shows the gap between practices in services marketing (we presented several examples in the introduction, such as Starbucks and Boots) and academic investigation about empathy training. This critical result is invisible in other systematic reviews on empathy training focusing solely on healthcare settings.  The second reason our paper adds value to the current systematic reviews on empathy is that we seize the absence of empirical papers investigating empathy training outside of healthcare settings as an opportunity to offer a research agenda to encourage service researchers to explore empathy training in service settings outside of healthcare organizations. There is no such discussion in the existing systematic review on empathy training. Besides, despite some differences between healthcare services and marketing services, both domains rely on users-employees interactions where social and empathic skills matter for service quality, user satisfaction, and service employees’ mental health. We provide more details about that matter in the revised discussion  Third, our systematic review focuses on empirical research implementing and testing the efficiency of empathy training in actual service interactions between service employees (non-students, adults, and frontline employees) and service users. In contrast, most published systematic reviews on empathy training include in-school programs and in-class simulations and do not consider service quality outcomes. Our intent to focus on actual service interactions is also consistent with the variables we thought to evaluate empathy training efficiency—perceived service quality, perceived service value, and user satisfaction—which is rarely discussed in previous systematic reviews.  Finally, we agree that our systematic review relies only on empathy training implemented in healthcare settings, and we have been transparent in the paper about this fact. However, we chose to keep loyal to our primary intention reflected in our search strategy and discuss our results through the prism of services. |
| The authors describe using the TIDieR checklist to report on the details of each individual studies, which is helpful. However, the authors do not comment on whether their systematic review is reported using any guideline, for example PRISMA. This would strengthen the reporting of the review and bring more clarity to why the review was done, what the authors did and what was found. | We used the PRISMA as required by Plos One’s policy and attached the PRISMA checklist as supporting information to our submission. |
| The authors identify a major limitation of the papers included, that only 50% include a definition of empathy. This is very important and it is good that the authors have included Table 3.  The authors however do not give their own definition of ‘empathy training’ in terms of their own inclusion criteria. The inclusion criteria includes ’at least one training in empathy is identifiable’ but it is unclear how ‘training in empathy’ is determined. As related terms were used in the search strategy (such as emotional intelligence or compassion), were studies included that the authors thought could be considered ‘empathy training’ but were in fact described as something else (e.g. active listening training)? Without a clear definition of what the authors considered to be ‘empathy training’ it would be difficult to say if the papers included were appropriate, particularly as approximately 50% of the papers included did not give a definition of empathy. Further clarity on how papers were included/excluded would be useful – for example were only studies that aimed to foster empathy as a primary outcome included, or were papers that aimed to improve communication skills, with a secondary outcome of fostering empathy also included? More clarity on the outcome variables would also be helpful in understanding how the authors made decisions on which papers should be included/excluded. | We thank the reviewer for this positive comment.  We also thank the reviewer for pointing out the absence of a definition for empathy training. We added the definition on page 5 (section 2.1). We also clarified in section 2.3 how we decided to include papers addressing empathy training through various terms and methods: “*We included only papers whose research objectives aim at testing a method (e.g., narrative training, role-play, mindfulness, communication training) to promote, foster, improve, or develop SEs’ empathic skills in service interactions.*” |
| Limitations of the selected papers (section 3.9) The first two sentences report a summary of the findings rather than limitations of the selected papers. It would be helpful for the reader to include a ‘summary of findings’ section and to summarise the findings of the review more clearly here. It would be helpful to include more details summarising each paper in table 2. The ‘limitations of selected papers’ section does not refer back to the MMAT scores that are discussed earlier in the paper. As the authors went to the trouble of using the tool, it would be helpful to see how the scores they arrived at using the tool link into their assessment of the selected papers limitations. The MMAT scores are described on page 14 under ‘results’ (section 3.1 Description of the included studies and methodological quality assessment) but more clarity on how papers were scored and what scoring involved would be helpful. Perhaps a table in the appendix showing the different MMAT domains and how each paper scored? | We thank the reviewer for this helpful comment. As suggested, we added another section (section 3.9. on page 23) to summarize the findings of our study. We also included more details outlining each paper in Table 2. We added the objective of the papers, provided more information for the sample section, added the duration of the training, and provided more details for the findings section (see Table 2, p. 11).  Regarding the MMAT, we added the tool as supporting information to the submission, showing all the domains—additional information about how the score was calculated was added (Methods section). In the Results section, we also added the score for each criterion per study design (available as an Appendix). We clearly identified which criterion was “poorly” reported in the study. |
| Discussion As the authors point out in the abstract, all 44 included papers focused solely on healthcare (predominantly nursing and physicians) and this significantly limits the generalizability of the findings in other sectors. However, in the discussion section (section 4, page 25-26) the authors suggest the findings can be generalised to other settings “empathy training programs identified…can be easily implemented and tested in other service settings”. This seems quite a leap of faith. | We understand the reviewer’s concern that our results might not be generalizable to other service settings. However, the results show that four primary skills were trained with empathic skills: communication and relationship building, emotional resilience, and counseling skills. These empathic skills have been discussed and praised in other service domains in business, and we cited several studies mentioning the importance of these skills when interacting with customers. Although the training content has to reflect the reality of the service settings and aims (e.g., scenarios, settings, expected outcomes, etc.), the four identified skills are perfectly relevant to service marketing as outlined in service research (e.g., Wieseke et al. 2012).  Besides, the reviewer refers to a sentence in our discussion that relate to the logistic of the empathy training, not the content. When we say that “*empathy training can be easily implemented in the workplace*,” we directly refer to the location, the duration, and the number of sessions. We assume that the flexibility of the logistics to implement empathy training programs, as identified in our systematic review, could benefit other service settings, such as service marketing. |
| The vast majority of the discussion section is taken up by discussion of the five main research areas the authors believe to be the top priority for future research in empathy training (table 5). There seems to be little connection between the findings of the systematic review (all of which are from a healthcare setting) and the future research priorities discussed. A clearer summary of what the main findings were and a more in-depth discussion of the findings would enhance the paper. | We thank the reviewer for this relevant comment. We rewrote the first part of the discussion, commented on the four empathic skills we identified in our systematic review, and connected them to service research. This new discussion is extended to 2 pages more than the previous version and enriched with 13 additional references from service research. |
| Conclusion This section includes a discussion on the limitations of the study. I would suggest that a separate heading for ‘limitations of the study’ would be helpful. The authors do report on some limitations, but do not report, for example on the heterogeneity of the papers included in the study. Another limitation of the study is that all the papers come from the same service field (healthcare). | We thank the reviewer for this helpful comment and changed the “conclusion” section into the “Limitations of the study” section. We also added the suggested limitations (p. 18). |
| The authors suggest that despite the limitations they describe, their study provides insightful information regarding empathy training programs in service research. It should perhaps be made clear here that the study provides insight to health service research only. | As explained above, we still believe that the empathy training programs identified in our study (i.e., communication and relationship skills, emotional resilience, and counseling skills) are relevant in service research since they deal with employee-user interaction and require the same type of empathic skills. We also raised the point that those specific skills were praised by prominent authors in the service research literature and resonate with industry practices in the service sector. Finally, we also outlined that the flexible implementation of empathy training programs (e.g., duration, location, etc.) makes them easily adaptable and implementable in other service settings. |
| The final section of the conclusion is describing the findings and might be better placed at the beginning of the discussion section. | We agree with the reviewer’s suggestion and placed the final section of the conclusion at the beginning of the discussion section. |

| **Reviewer #2** | **Responses** |
| --- | --- |
| The authors have done invaluable work to gather current evidence on empathy training (mainly health simulation) and to see whether this is similarly applied in a different field (i.e service industries in business studies). Their rational for the need of empathy training in service industries is sound. The literature review approach is carried out thoroughly and transparent. | We thank the reviewer very much for this positive feedback. |
| Their suggestions for further research are an excellent starting point for further research. I dare to say that further fields could benefit from empathy training such as education, leadership and social care (which is partly included in the health simulation). | We thank the reviewer for this comment. |
| The areas for some improvements for me are around how the authors explain understand empathy – how it differs from compassion, sympathy and empathic concern. | We thank the reviewer for suggesting this improvement. We added a paragraph explaining how empathy, empathic concern, and prosocial behaviors interact (see page 30). |
| I further would like to see more explanation “on empathy is on a one way process” and social interactions… having said this authors provide an explanation for service encounters (albeit a slightly old reference Siehl et al 1992), yet I would appreciate a few more positions on this (e.g. Luhman, Sacks, Geoffman, Silverman – I’m just noticing that these would also be ‘old’) | We thank the reviewer for suggesting this improvement. We extended and improved the paragraph explaining that empathy is a two-way process, meaning that users’ emotions affect employees’ emotional states, who respond to the users with these emotions (see page 31). |
| Are the service encounters always expected to be one to one? How is ‘empathy’ exchange process in a triad or larger group? | We consider that service interactions are mainly based on a one-on-one basis. Most of the literature in service refers to one-to-one interactions (e.g., Wieseke et al., 2012). However, the reviewer is correct when some service interactions may imply more than two people. For instance, it is the case in hospitality and tourism (e.g., restaurants, hotels, etc.) when one service provider interacts with a group of customers. However, even in service interaction with a group of customers, the service provider would interact with one person at a time, which will reproduce a one-to-one conversation setting. |
| All in all I really enjoyed reading this manuscript and would like to thank the authors. | We thank the reviewer for this comment. |
